# Ballistic Simulation of a Head Wound Inflicted by a Homemade Gun Used in a Suicide Case

**DOI:** 10.7759/cureus.22145

**Published:** 2022-02-12

**Authors:** Nikolaos Tsiatis, Konstantinos Katsos, Emmanouil Sakelliadis, Chara Spiliopoulou, Konstantinos Moraitis

**Affiliations:** 1 Department of Forensic Medicine and Toxicology, National and Kapodistrian University of Athens, School of Medicine, Athens, GRC

**Keywords:** wound ballistics, experimental shooting, homemade gun, gunshot wound, forensic science, forensic medicine

## Abstract

Homemade guns may inflict serious injuries mainly depending on the shooting distance. A case of a male victim discovered dead with a head wound thought to be inflicted by a homemade gun firing modified ammunition is presented. Upon completion of the postmortem examination, the question of whether the homemade gun recovered on site was able to inflict such a wound arose. An experimental approach was employed to evaluate the ballistic characteristics and wounding pattern of the homemade gun and then compare it with the actual case. Ballistic gelatin with an incorporated bone simulant was used to model and approximate the behavior of the projectile when striking and penetrating the victim’s head. The retention of the bullets’ path in gelatin was verified through experimental shootings using the same homemade gun and similar type of projectiles. Analysis of the experimental shootings allowed for improved observation and documentation of the wounding pattern, thus confirming the initial hypothesis that the recovered homemade gun did in fact inflict the wound observed during the autopsy of the victim.

## Introduction

Firearm acquisition, possession, or transfer is restricted by Greek legislation to licensed individuals, according to Law No. 2168/1993 as amended by Law No. 4678/2020. Applicants for a gun license need to provide a genuine reason to possess a firearm, for example, hunting, practical shooting, or personal protection. Thus, homemade guns that may be easily constructed may emerge as “firearm alternatives.” The variability of these alternatives is limited only by the constructor’s ability [1]. It seems that almost anyone may construct a homemade gun merely by following some relatively simple instructions [2].

Homemade guns are mainly constructed either artisanally or by a handcrafted process. In many cases, they may not even have the typical gun shape (e.g., stud guns, self-modified shotgun, and veterinary guns) [3]. Therefore, many are usually obsolete, improvised, non-branded, substandard firearms, which differ from classic firearms both in appearance and in ballistic behavior. It appears that their construction cost is lower compared to standard modern firearms manufactured in factories. Their use often leads to unpredictable and misleading reconstruction of the shooting incident due to the lack of class characteristics marks that link the bullets fired from smoothbore firearms and their wounding effectiveness [4].

Experimental shootings into tissue simulant materials are essential to evaluate the behavior of a projectile in wound ballistics. As relevant literature is limited, the bullet’s path inside the human body following skin perforation is rather difficult to predict. Thus, ballistic gelatin may prove to be quite useful in the documentation of the wound channel. Tissue simulant materials are known to reflect the transfer of the bullet’s kinetic energy after penetration. More specifically, after penetration and during the deceleration of the projectile, the medium is stretched perpendicularly to the projectile’s course, thus leading to a “temporary cavity” formation [5]. Many factors determine the damage extent of the tissue surrounding the bullet’s path, such as gun and caliber type, the bullet’s shape, construction and design, tissue density, and elasticity energy transferred to the tissues [6].

In this study, a ballistic approach based on a fatal case is presented, in which a homemade gun with modified ammunition was used.

## Case presentation

An 81-year-old male was discovered dead, in a supine position, outside his house in an open field area, bearing a penetrating gunshot wound to the head. Blood had flowed from the head wound and from the nasal cavity. A rusty gun-shaped device and an unspent cartridge were discovered during police investigation in close proximity to the body. Due to the location of the scene in an open field area, the fatality-causing bullet was not recovered. A suicidal note was also found on the clothes that included statements relevant to a three-year plan to commit suicide and forgiveness requests from anyone the deceased might had unintentionally hurt in the past. His relatives reported that he had suffered from depression for some period without the use of associated prescribed medication. Based on the evidence collected at the scene, the death was classified by police authorities as a possible suicide.

Autopsy findings

**Figure 1:**
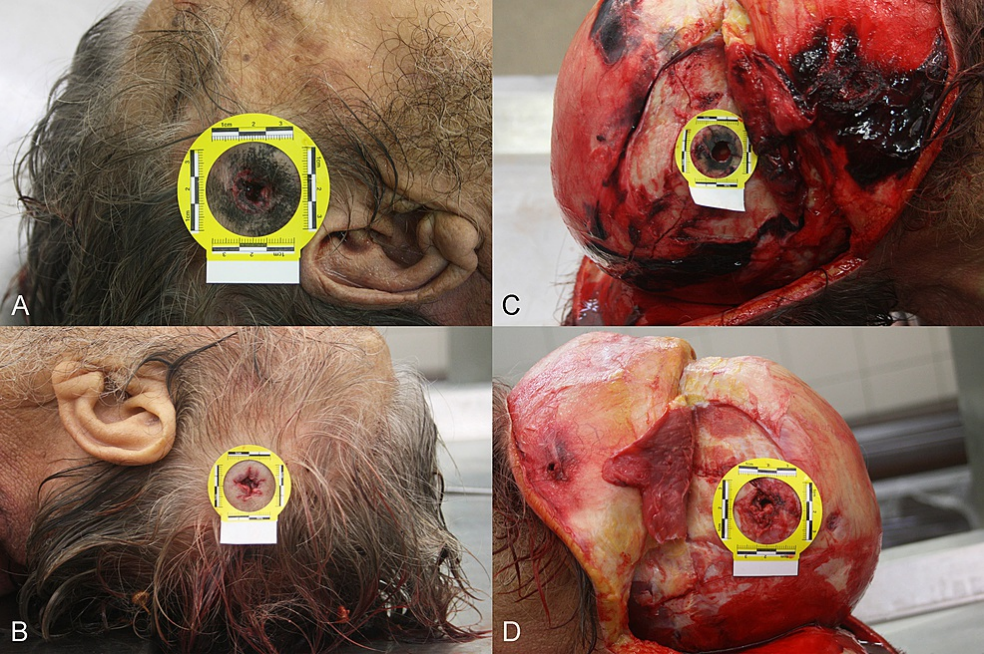
(Α) The entrance wound with soot deposition surrounding the wound edge and a muzzle imprint. (B) The exit wound. (C) The entrance wound after dissection of the soft tissues. (D) External beveling of the outer table of bone at the exit wound.

A penetrating gunshot wound to the head was noted during the external examination. The entrance wound, measuring 13mm in diameter, with burned-intruding margins, was located on the right temporal region approximately 35mm from the upper limit of the ear (Figure 1A). An area of soot deposition surrounded the wound edge, and a muzzle imprint was noted. The exit wound, measuring 10mm in diameter, was located on the left temporal region (Figure 1B). It presented irregular and protruding margins. Furthermore, bilateral periocular bruises were noted. No other external injuries were observed.

Upon dissection of the head soft tissues, soot deposition was observed on the right temporal bone surrounding the opening (Figure 1C), while typical internal beveling of the inner table of the bone was also present. Based on these characteristics, it was concluded that the wound observed on the right temporal region was inflicted while the muzzle of the gun was placed in loose contact with the head during the discharge of the firearm. On the left temporal bone, an external beveling of the outer table was evident, while soot deposition was absent (Figure 1D).

Macroscopic examination of the brain revealed a cavity extending between the temporal lobes. Fractures of the skull base were also observed, causing the bilateral periocular bruises. No other internal injuries were observed. Macroscopic examination of the heart revealed a myocardial scar at the interventricular septum, as well as coronary artery disease (50% stenosis of the left anterior descending branch of the left coronary artery). The renal surfaces were diffusely microgranular (lesion suggestive of hypertension). The rest of the organs did not reveal any significant pathology. Toxicological analysis revealed that the deceased was not under the influence of any psychotropic substances.

Death was attributed to craniocerebral injuries due to a single gunshot wound to the head. In order to assess the functionality and the ability of the recovered firearm to cause fatal injuries, a ballistic examination and experimental shots were conducted with the same weapon.

Ballistic examination

**Figure 2:**
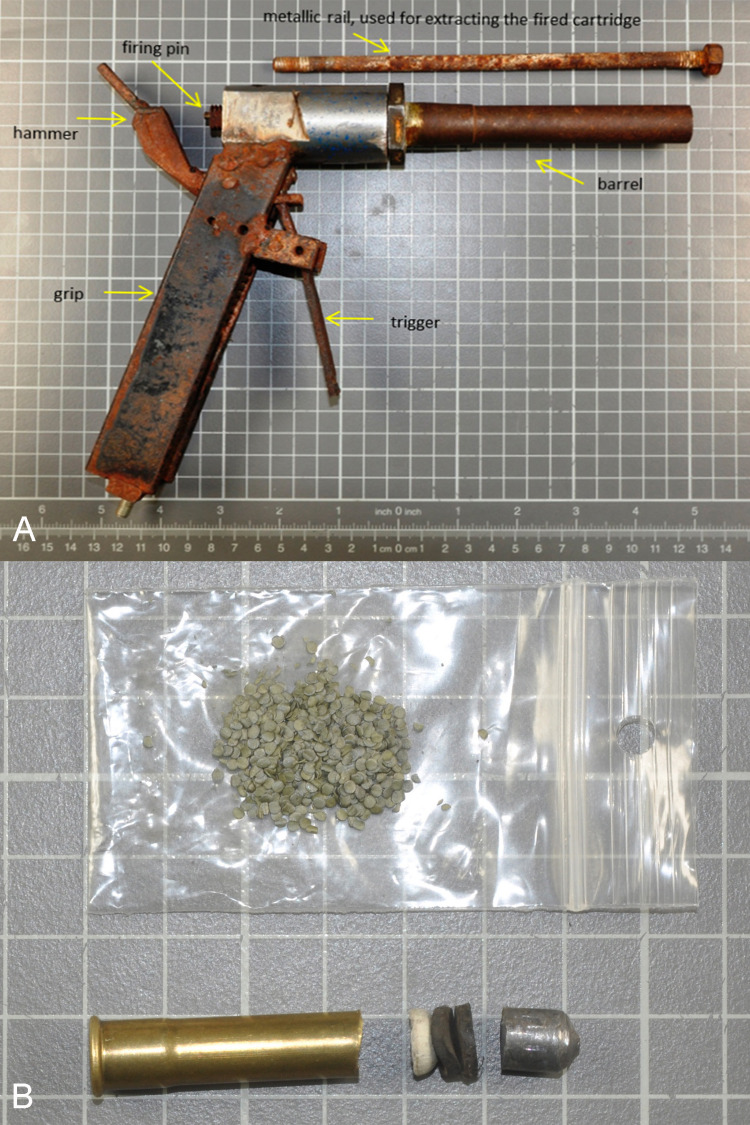
(A) The homemade gun. (B) The modified 9mm Flobert cartridge recovered at the scene (disassembled).

Examination of the recovered firearm revealed that it had been constructed with great attention to detail. Its parts consisted of iron pieces crafted and welded together (Figure 2A). The gun was found in a rusted state, probably due to improper storage conditions for a long time. Its barrel had a smooth bore with no rifling and measured 145mm in length, including the chamber. The internal diameter of the front end was 8-9mm. The chamber’s internal diameter was 9mm.

A 9mm Flobert caliber cartridge case was found within the chamber. Another unspent cartridge was found near the victim’s body. When disassembled, it revealed that it was derived from a manufactured 9mm Flobert cartridge, which had been converted as its shotshells were replaced with a homemade lead bullet (weight: 4.38g; diameter: 7.9mm; length: 9.6mm) (Figure 2B). Between the powder and the bullet, a cylindrical piece of felt and two pieces of rubber were impacted, obviously to increase the gas pressure after firing (Figure 2B).

Experimental technique

To carry out experimental shootings, four manufactured 9mm Flobert cartridges were modified, disassembled, and then assembled following the abovementioned procedure to achieve similarity with the cartridge recovered at the scene. As homemade guns are not properly manufactured and tested, all necessary measures were taken in order to conduct the experiments in a safe manner. Protective gloves were used to grip the gun, as well as a Kevlar fiber cuff to cover the shooter’s arms. In addition, bulletproof glass was placed in front of the shooter, and safety glasses and earplugs were worn during the experimental shootings.

Test shots were fired into ballistic gelatin to model and approximate the behavior of the modified bullet while striking and perforating the victim’s head. For this study, ballistic gelatin blocks were prepared using Gelita® Gelatin Type Ballistic 3 (Gelita AG, Eberbach, Germany). In order to create ideal simulation conditions, the ballistic gelatin used was produced into a mold. A bone model was placed into the mold. This model was a Synbone® “Generic hollow hemi-sphere 5mm” modified bone-like polyurethane, with a diameter of 190mm, wall thickness of 5mm, and weight of 215g ± 5% (Synbone AG, Zizers, Switzerland).

**Figure 3:**
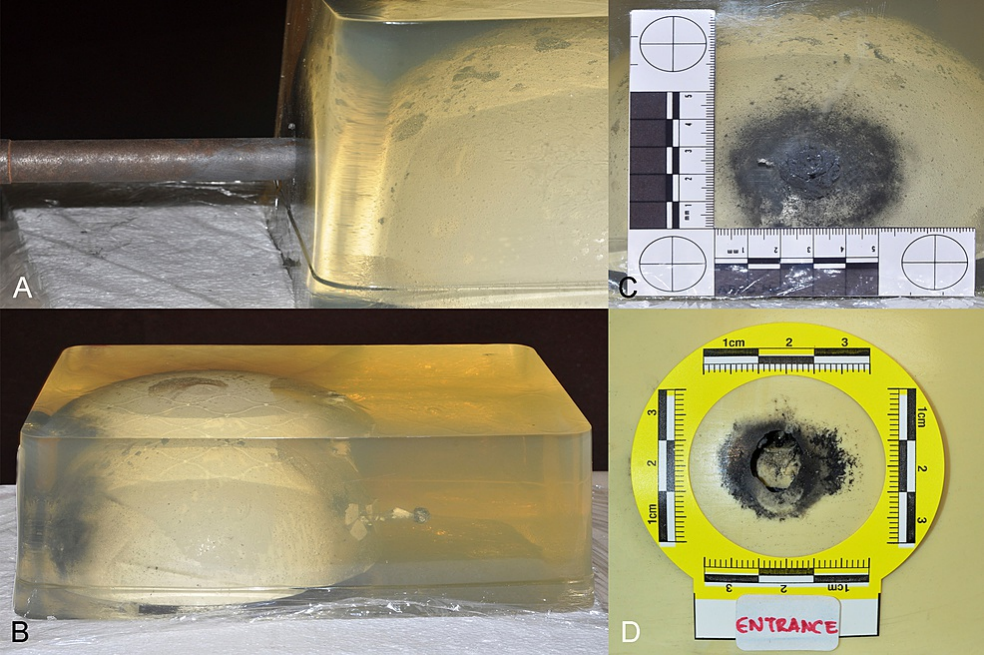
(A) Contact test shooting with the handmade gun. (B) The bullet path through the gelatin and bone model (from left to right). (C–D) Soot deposition surrounding the contact point on the gel and bone model at the bullet’s entrance.

Considering that the soot deposition surrounded the victim’s entrance wound, two test shots were fired in contact with the gelatin using the same homemade gun and modified ammunition, with a lead bullet weighing out 4.38g (67.6 grains) (Figure 3A). Temperature (14.3°C/57.8°F) and humidity (34%) were measured during the experimental shootings. The purpose of the test shootings was to calculate the kinetic energy of the projectile to ascertain the characteristics of the entrance/exit wound and to record the bullet’s path.

Impact velocity (V_imp_) was determined through a chronograph placed at the appropriate distance (almost 30cm) in front of the gun’s muzzle. The initial kinetic energy for the linear motion translation of the bullet was calculated as E_o _= 1/2 m V^2^, where (m) is the bullet’s mass (kg), (V) is the bullet’s impact velocity (m/seconds), and (E_o_) is kinetic energy (Joule = kg m^2^/second^2^). Data collected from these experimental shots are presented in Table 1. The impact velocity was measured (on average) close to 370m/second (1215ft/second), and the kinetic energy was calculated close to 300 Joules.

**Table 1:** Data from two shots for calculating the initial energy (Eo) of the bullet.

	First shot	Second shot
V_imp_ (m/second)	385.6	355.1
V_imp_ (ft/second)	1265.09	1165.02
m (g)/(grains)	4.38/67.6	
E_o_ (Joule)	325.62	276.15

The block was photographed after the shooting. Bullets entered the bone model, then kept almost a straight line moving through the gel, and exited simulating the bullet path observed at autopsy (Figure 3B). Soot deposition surrounded the entrance contact point both on the gel and on the bone model (Figures 3C, 3D).

**Figure 4:**
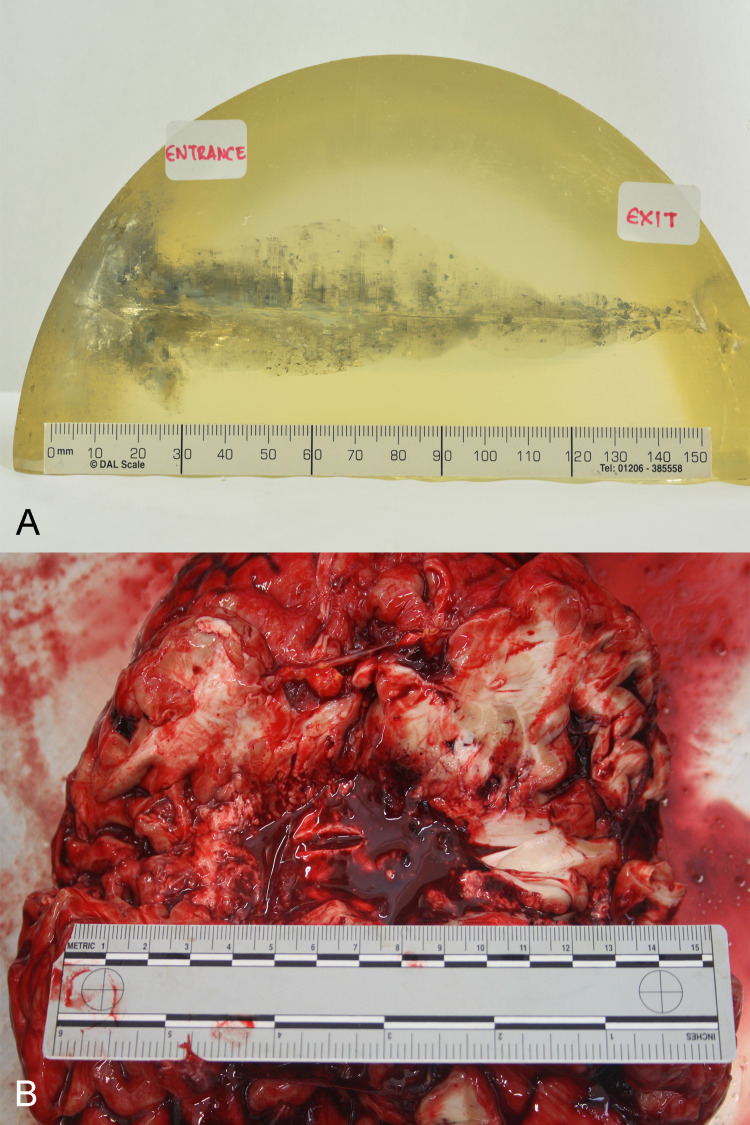
(A) The created permanent cavity into the ballistic gelatin. (B) Similar damage observed on the victim’s brain parenchyma.

Both bullets’ movements were similar, with both leading to the creation of a permanent cavity. Their paths nearly followed the shooting line up until their exit from the bone model. The created cavity was greater in dimensions close to the entrance point and gradually weakened toward the exit (Figure 4A). A similar cavity was observed at the autopsy of the deceased during the macroscopical examination of the brain parenchyma (Figure 4B).

**Figure 5:**
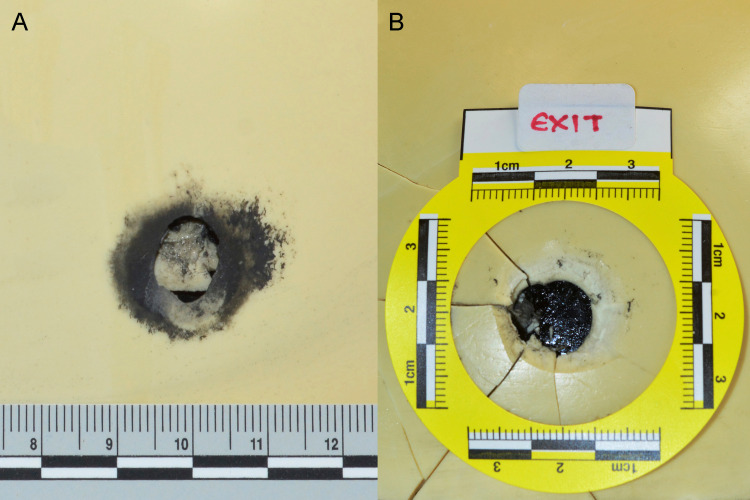
(A) The entrance opening on the bone model. (B) The exit opening with typical external beveling and radiating fractures.

In both shooting tests, the bullets used produced entrance and exit holes measuring 9-10mm and 11-12mm, respectively. The deviation of the bullets’ initial paths while moving through the block measured 0.4°, remaining slightly closer to the original shooting line. In general, the test results verified the behavior of the bullet within the victim’s head (Figure 5).

The microscopic examination of the test-fired cartridges matched with the recovered cartridge case, thus identifying the reputed gun. Due to the smoothbore barrel of the homemade gun, no individual markings were produced on the test-fired bullets for further examination.

## Discussion

Homemade guns may be unsafe and dangerous for any user, and it is rather uncommon to have been tested before they are first used. Literature on this subject is scarce but has grown over the last years [3]. There is a wide variation about the potential purposes of homemade guns, which may include protection, self-defense, homicide, sport, illegal hunting, and finally suicide [7]. Nonetheless, globally, cases where a homemade firearm is used to perform a suicide are exceedingly rare [8]. Furthermore, homicides and accidents with homemade weapons are extremely uncommon [9]. It should be noted that according to a 1995 survey in Canada, 28% of students who responded admitted to carrying a weapon at school, which was often a homemade one (11.6% among weapons reported) [10].

In India, these guns are popularly known as country-made firearms [4]. In the United States, they are called “zip guns” designated as either crude homemade firearms or coming from the conversion of a blank/tear gas cap gun to a firearm. They were popular in the 1950s mainly in urban areas due to the construction easiness and the availability of materials/parts [11]. Nowadays, homemade guns are less commonly found in Western Europe, Australia, and the United States but are still being used in criminal cases in India, South Africa, and Israel [12]. In Japan, a country that has far fewer cases of injury or death by firearms than by other weapons, only a few suicide cases using homemade firearms have been reported [13].

Homemade firearms, as a rule, are smoothbored. Commercial ammunition mostly for small caliber rifle handguns (pistols and revolvers) or for shotguns is used. Shooting with homemade guns may not be efficient, particularly in shooting from a distance due to the absence of spinning missiles in smoothbore barrels. In general, such bullets present low velocity and energy; thus, their wounding effectiveness is consequently low. Nevertheless, when shots are fired in contact or from a close range, they may cause serious or even fatal injuries [1]. Each case presents its individual characteristics. For example, the morphology of the entrance wounds depends mainly on the energy transfer of the projectile, as well as on the barrel length, the caliber of the bullet, and the cartridge powder load.

Homemade guns may be constructed from easily accessible materials. Frequently, they can be fashioned from pieces of metal, metallic cylinders for the barrel, and nails for the firing pin or using a piece of wood for the grip [11]. Nevertheless, possibly due to the artisanal assembly method, the creation of a wide-enough exit for the gunshot gases to expand is rather problematic. In the presented case study, the soot deposition surrounding the entry opening on the bone (3-4cm in diameter) may also be related to these “construction” issues. The same effect is observed with the blank cartridge guns that produce great gas pressure at their muzzle after firing. Should this type of gun be placed in contact with the surface of the human body, it can cause destruction of the skin and underlying structures including bones [14,15]. Concerning homemade guns, several reports cite serious injuries and even fatalities due to modified blank cartridge guns. Upon simple modifications such as removing the barrier that exists into the barrel, these blank cartridge guns gain the ability to propel small spheres placed inside the cartridge or a homemade projectile inserted into the edge of the cartridge [16]. A lot of homemade devices/firearms use either a standard sprung hammer or trigger mechanisms. In the reported cases, when an unusual and cumbersome nature of the firing mechanism is mentioned, it is likely that these homemade devices were specifically manufactured for suicidal purposes [12].

In homemade guns, the entrance wounds may present both different characteristics and morphology, depending mainly on the shooting distance. Contact or very close-range shots will produce an entrance wound on the skin surrounded by a muzzle imprint. The skin may rupture in a starlike pattern resulting from gas pressure buildup [15]. Furthermore, ammunition and entrance wound dissociation, extensive bruising around the entrance wound, and unexpected smoothening could be specific signs in the forensic assessment of handmade gun wounds [3]. Proper wound documentation during the autopsy, as well as any subsequent efforts to reconstruct a shooting, is of paramount importance in forensic pathology. Experimental shooting may actually provide further insight into the bullet’s behavior within the human body, especially in some demanding cases.

The results obtained from the ballistic examination of the gun and the experimental shots fired with the same weapon were critical to the evaluation of the death and its circumstances. The ballistic examination determined that this homemade gun was functional and able to cause the specific findings (injuries and soot deposition). Through test shootings in ballistic gelatin, it was feasible to approximate the effects of a specific bullet both on the skull and on the brain parenchyma. From the measured impact velocities, it was proved that the death was caused by a bullet transferring a high quantity of energy (close to 300 Joule). Should further characterization of the experimental wound ballistics be required, computed tomography technology may also be employed.

## Conclusions

Homemade guns used as “firearm alternatives” may inflict serious injuries depending on the shooting distance and the anatomical region affected. In suspected suicides committed with a homemade gun, a ballistic examination and experimental shots can be conducted with the same weapon and similar type of projectiles to assess the functionality and the ability of the recovered firearm to cause fatal injuries. As presented in this case, ballistic gelatin with an incorporated bone model can be used to simulate head wounds inflicted by homemade guns. The obtained results affirmed that the higher the impact velocity of the projectile, the further the distance of the bullet path in ballistic gelatin as in a human gunshot victim. The terminal ballistic behavior and penetration of modified bullets in gelatin can closely approximate what happened in living tissue.

## References

[1] Gojanović MD (1995). Fatal firearm injuries caused by handmade weapons. J Clin Forensic Med.

[2] Demirci S, Gunaydin G, Dogan KH, Erkol Z (2008). Deaths caused by mole guns: three case reports. Int J Legal Med.

[3] Chérrez Bermejo C, Teijeira Álvarez R (2018). A quite rare case of bruising abrasion-like as gun entry wound. Forensic Sci Int.

[4] Sinha JK (2015). Forensic investigation of unusual firearms: ballistic and medico-legal evidence.

[5] Schyma C, Madea B (2012). Evaluation of the temporary cavity in ordnance gelatine. Forensic Sci Int.

[6] Korać Z, Kelenc D, Baskot A, Mikulić D, Hancević J (2001). Substitute ellipse of the permanent cavity in gelatin blocks and debridement of gunshot wounds. Mil Med.

[7] Zdarilek M, Nevická E, Rozboril R, Ťažký B, Šidlo J (2017). A suicidal gunshot injury with a homemade zip gun during a traffic stop. Rom J Leg Med.

[8] Le Garff E, Delannoy Y, Mesli V, Berthezene JM, Morbidelli P, Hédouin V (2015). Homemade firearm suicide with dumbbell pipe triggering by an air-compressed gun: case report and review of literature. Am J Forensic Med Pathol.

[9] Hejna P, Safr M (2010). An unusual zip gun suicide--medicolegal and ballistic examination. J Forensic Sci.

[10] Dandurand Y (1998). Firearms, accidental deaths, suicides and violent crime: an updated review of the literature with special reference to the Canadian situation. Vancouver, Canada: International Centre for Criminal Law Reform and Criminal Justice Policy.

[11] Cunliffe CH, Denton JS (2008). An atypical gunshot wound from a home-made zip gun--the value of a thorough scene investigation. J Forensic Sci.

[12] Hartwig S, Tsokos M, Schmidt S, Byard RW (2010). Self-constructed shooting devices utilizing manually-impacted firing-pins (suicide machines). Am J Forensic Med Pathol.

[13] Tsuboi A, Satoh F, Seto Y, Osawa M (2014). Self-inflicted fatal shotgun wound from a homemade weapon. Leg Med (Tokyo).

[14] Buyuk Y, Cagdir S, Avsar A, Duman GU, Melez DO, Sahin F (2009). Fatal cranial shot by blank cartridge gun: two suicide cases. J Forensic Leg Med.

[15] Demirci S, Dogan KH, Koc S (2011). Fatal injury by an unmodified blank pistol: a case report and review of the literature. J Forensic Leg Med.

[16] Uzün I, Büyük Y, Erkol Z, Ağritmiş H, Kir Z (2009). Fatalities caused by spherical bullets fired from blank cartridge guns in Istanbul, Turkey. J Forensic Sci.

